# Changes in Emergency Department Pediatric Readiness and Mortality

**DOI:** 10.1001/jamanetworkopen.2024.22107

**Published:** 2024-07-22

**Authors:** Craig D. Newgard, Shauna Rakshe, Apoorva Salvi, Amber Lin, Jennifer N. B. Cook, Marianne Gausche-Hill, Nathan Kuppermann, Jeremy D. Goldhaber-Fiebert, Randall S. Burd, Susan Malveau, Peter C. Jenkins, Caroline Q. Stephens, Nina E. Glass, Hilary Hewes, N. Clay Mann, Stefanie G. Ames, Mary Fallat, Aaron R. Jensen, Rachel L. Ford, Angela Child, Brendan Carr, Kendrick Lang, Kyle Buchwalder, Katherine E. Remick

**Affiliations:** 1 Center for Policy and Research in Emergency Medicine, Department of Emergency Medicine, Oregon Health & Science University, Portland; 2 Knight Cancer Institute Biostatistics Shared Resource, Oregon Health & Science University, Portland; 3 Los Angeles County Emergency Medical Services, Harbor-UCLA Medical Center, Torrance, California; 4 Department of Emergency Medicine, University of California, Davis School of Medicine, Sacramento; 5 Department of Pediatrics, University of California, Davis School of Medicine, Sacramento; 6 Centers for Health Policy, Primary Care and Outcomes Research, Department of Medicine, Stanford University School of Medicine, Palo Alto, California; 7 Division of Trauma and Burn Surgery, Center for Surgery Care, Children’s National Hospital, Washington, DC; 8 Department of Surgery, Indiana University School of Medicine, Indianapolis; 9 Department of Surgery, University of California, San Francisco, San Francisco; 10 Department of Surgery, Rutgers New Jersey Medical School, Newark; 11 Department of Pediatrics, University of Utah School of Medicine, Salt Lake City; 12 Department of Surgery, University of Louisville School of Medicine, Norton Children’s Hospital, Louisville, Kentucky; 13 Department of Surgery, University of California, San Francisco, Benioff Children’s Hospital, San Francisco; 14 Oregon Emergency Medical Services for Children Program, Oregon Health Authority, Portland; 15 Department of Emergency Medicine, Icahn School of Medicine at Mount Sinai, New York, New York; 16 Department of Pediatrics, Dell Medical School, University of Texas at Austin, Austin; 17 Department of Surgery, Dell Medical School, University of Texas at Austin, Austin

## Abstract

**Importance:**

High emergency department (ED) pediatric readiness is associated with improved survival, but the impact of changes to ED readiness is unknown.

**Objective:**

To evaluate the association of changes in ED pediatric readiness at US trauma centers between 2013 and 2021 with pediatric mortality.

**Design, Setting, and Participants:**

This retrospective cohort study was performed from January 1, 2012, through December 31, 2021, at EDs of trauma centers in 48 states and the District of Columbia. Participants included injured children younger than 18 years with admission or injury-related death at a participating trauma center, including transfers to other trauma centers. Data analysis was performed from May 2023 to January 2024.

**Exposure:**

Change in ED pediatric readiness, measured using the weighted Pediatric Readiness Score (wPRS, range 0-100, with higher scores denoting greater readiness) from national assessments in 2013 and 2021. Change groups included high-high (wPRS ≥93 on both assessments), low-high (wPRS <93 in 2013 and wPRS ≥93 in 2021), high-low (wPRS ≥93 in 2013 and wPRS <93 in 2021), and low-low (wPRS <93 on both assessments).

**Main Outcomes and Measures:**

The primary outcome was lives saved vs lost, according to ED and in-hospital mortality. The risk-adjusted association between changes in ED readiness and mortality was evaluated using a hierarchical, mixed-effects logistic regression model based on a standardized risk-adjustment model for trauma, with a random slope–random intercept to account for clustering by the initial ED.

**Results:**

The primary sample included 467 932 children (300 024 boys [64.1%]; median [IQR] age, 10 [4 to 15] years; median [IQR] Injury Severity Score, 4 [4 to 15]) at 417 trauma centers. Observed mortality by ED readiness change group was 3838 deaths of 144 136 children (2.7%) in the low-low ED group, 1804 deaths of 103 767 children (1.7%) in the high-low ED group, 1288 deaths of 64 544 children (2.0%) in the low-high ED group, and 2614 deaths of 155 485 children (1.7%) in the high-high ED group. After risk adjustment, high-readiness EDs (persistent or change to) had 643 additional lives saved (95% CI, −328 to 1599 additional lives saved). Low-readiness EDs (persistent or change to) had 729 additional preventable deaths (95% CI, −373 to 1831 preventable deaths). Secondary analysis suggested that a threshold of wPRS 90 or higher may optimize the number of lives saved. Among 716 trauma centers that took both assessments, the median (IQR) wPRS decreased from 81 (63 to 94) in 2013 to 77 (64 to 93) in 2021 because of reductions in care coordination and quality improvement.

**Conclusions and Relevance:**

Although the findings of this study of injured children in US trauma centers were not statistically significant, they suggest that trauma centers should increase their level of ED pediatric readiness to reduce mortality and increase the number of pediatric lives saved after injury.

## Key Points

**Question:**

Were changes in emergency department (ED) pediatric readiness from 2013 to 2021 associated with changes in pediatric mortality at trauma centers?

**Findings:**

In this cohort study of 467 932 injured children at 417 US trauma centers, observed mortality was highest among EDs with persistently low pediatric readiness. Using a risk-adjustment model, high-readiness EDs (persistent or change to) had 643 additional lives saved, whereas low-readiness EDs (persistent or change to) had 729 additional preventable deaths.

**Meaning:**

Changes to ED readiness among US trauma centers were associated with pediatric mortality, lives saved, and lives lost, depending on the direction of change; although the findings were not statistically significant, the results suggest that trauma centers should increase their level of pediatric readiness to decrease mortality.

## Teaser

This cohort study examines the association of changes in emergency department pediatric readiness with pediatric mortality at US trauma centers between 2013 and 2021.

## Introduction

The National Pediatric Readiness Project (NPRP) is a national quality improvement initiative created more than a decade ago to ensure that all emergency departments (EDs) have the readiness (ie, care coordination, personnel, quality improvement, safety, policies and procedures, and equipment) to care for acutely ill and injured children.^1^ As part of the NPRP, national assessments of ED pediatric readiness were completed in 2013 and 2021.^2,3^ According to the 2013 assessment, a high level of ED pediatric readiness is associated with improved survival among critically ill children,^4^ injured children admitted to trauma centers,^5,6^ and children with acute injuries or medical illness requiring hospitalization.^7^ The only outcomes-based study^8^ using the 2021 NPRP data showed that low ED readiness was associated with worse survival in trauma centers.

Among EDs across the US, ED pediatric readiness decreased from 2013 to 2021, primarily due to the loss of pediatric emergency care coordinators (PECCs).^3^ However, changes in ED pediatric readiness among US trauma centers have not been described, to our knowledge, and the impact of such changes remains unknown. Because assessment of ED pediatric readiness was integrated to the 2022 American College of Surgeons trauma center verification criteria,^9^ understanding the impact of changes to ED readiness may help guide trauma centers and help address the recent national increase in childhood mortality.^10^

In this study, we examined changes in ED pediatric readiness among US trauma centers, as measured in 2013 and 2021, and the association with pediatric mortality (ED and in-hospital). We quantified the mortality associated with changes in ED pediatric readiness using the number of pediatric lives saved vs lives lost among trauma centers over a 10-year period.

## Methods

### Study Design

This was a retrospective cohort study that was reviewed and approved by institutional review boards at Oregon Health and Science University and the University of Utah School of Medicine. The institutional review boards waived the requirement for informed consent because the study was based on existing data that were previously collected, consistent with 45 CFR §46. We used the Strengthening the Reporting of Observational Studies in Epidemiology (STROBE) reporting guidelines for cohort studies.^11^

### Study Setting

We included trauma centers (levels I to V, adult and pediatric) in 48 states and the District of Columbia that submitted pediatric data to the National Trauma Data Bank (NTDB) from 2012 through 2021 and completed the 2013 and 2021 NPRP assessments. We created 2 analytic samples: (1) a hospital-level sample of 716 trauma centers completing both national assessments, regardless of availability of patient-level data and (2) a patient-level sample of injured children in 417 trauma centers that completed both national assessments and submitted data to NTDB from 2012 through 2021. To be inclusive and reduce selection bias, we included 109 trauma centers (26% of the 417 hospitals) that submitted at least 1 year of data from 2012 to 2014 and from 2019 to 2021; most of the 109 hospitals were missing only 1 to 2 years of data. The NTDB data are collected using standardized inclusion criteria and include detailed information from the initial ED presentation and hospital stay, including mortality.^12^

### Patient Population

For the patient-level sample, we included injured children younger than 18 years meeting the NTDB inclusion criteria of an injury diagnosis with hospital admission, transfer, or injury-related death in a participating trauma center from January 1, 2012, through December 31, 2021. We included patients classified as dead on arrival on the basis of previous research showing that a portion of these children survive.^5^ For children transferred to other trauma centers, we matched available records from the second hospital using probabilistic linkage.^13^ We excluded children missing the initial ED record, transferred without a linked record from the second hospital, missing final hospital disposition, treated in nontrauma centers, or treated in EDs without 2 matched NPRP assessments (eFigure 1 in Supplement 1). To allow for model convergence, we also excluded EDs treating fewer than 10 pediatric trauma patients per year.

We prespecified 3 subgroups of seriously injured children likely to be sensitive to ED pediatric readiness: Injury Severity Score (ISS; range, 0-75, with higher scores denoting worse injuries) 16 or higher,^14,15,16^ head Abbreviated Injury Scale (AIS) score (range, 0-6, with higher scores indicating worse injuries) 3 or higher (serious brain injury), and children requiring early critical resources.^17^ We defined early critical resources according to a consensus definition for children^17^ that included any of the following within 24 hours of ED presentation: airway management, blood transfusion, nonorthopedic surgery (including intracranial pressure monitoring), vasopressor support, extracorporeal membrane oxygenation, or presence of spinal cord injury (eTable 1 in Supplement 1).

### ED Pediatric Readiness

The primary exposure was the change in ED pediatric readiness, measured using the weighted Pediatric Readiness Score (wPRS) in the 2013 and 2021 NPRP assessments from the initial ED.^2,3^ The NPRP assessments included US EDs providing emergency care 24 hours per day, 7 days per week. The 2013 assessment was completed from January through August 2013 (83% response rate, 4146 of 5017 invited hospitals).^2^ The 2021 assessment was completed from May through August 2021 (71% response rate, 3557 of 5150 invited hospitals).^3^ There were 2825 US hospitals that responded to both assessments.^3^ The wPRS is a weighted score from 0 to 100 based on questions with moderate to high clinical importance in 6 domains,^18^ with 100 representing the highest level of ED pediatric readiness.^2,3^ The 2013 and 2021 assessments used the same domains of readiness and a similar weighting scheme to generate the wPRS. We linked the NPRP assessment to the initial trauma center ED using hospital name, address, and zip code.

We defined high ED pediatric readiness as wPRS 93 or higher, a level associated with increased survival in US trauma centers,^6,8^ vs low readiness (wPRS <93). On the basis of the level of ED readiness in 2013 vs 2021, we categorized trauma centers into 4 categories: high-high (persistently high ED readiness), low-high (change from low to high ED readiness), high-low (change from high to low ED readiness), and low-low (persistently low ED readiness).

### Variables and Outcomes

Patient-level variables included demographic characteristics (age, sex, race, and ethnicity, as abstracted from the medical record), comorbidities, initial ED systolic blood pressure and Glasgow Coma Scale score (range, 3-15 with higher scores denoting better neurologic function), emergent airway intervention, mechanism of injury, mode of arrival, AIS score,^15^ ISS,^14,15^ hospital procedures, blood transfusion, interhospital transfer, and length of hospital stay. Race and ethnicity are included in this study because they have been associated with differences in mortality across levels of ED pediatric readiness among injured children.^19^ To maximize information on ED and hospital procedures, we used a combination of abstracted NTDB data fields and *International Classification of Diseases, Ninth Revision *or* International Statistical Classification of Diseases and Related Health Problems, Tenth Revision* procedure codes, categorized using the Agency for Healthcare Research and Quality Clinical Classification System.^20^ We then mapped Clinical Classification System categories to standardized operative domains, airway management, and blood transfusion.

To define trauma center level (I to V) and type (adult and pediatric), we used the most recent data (2021) and the following order of data sources: American College of Surgeons verification status, state-level designation, NPRP assessment, and the annual American Hospital Association survey. We also created hospital-level variables for annual ED pediatric volume and pediatric trauma volume. The primary outcome was the number of additional lives saved vs lives lost (including ED and in-hospital deaths) based on changes to ED pediatric readiness.

### Statistical Analysis

Data analysis was performed from May 2023 to January 2024. We compared observed (unadjusted) mortality in the 4 ED pediatric readiness change groups using Bonferroni-corrected pairwise comparisons.^21^ To evaluate the risk-adjusted association between changes in ED readiness and mortality, we used a hierarchical, mixed-effects logistic regression model based on a standardized risk-adjustment model for trauma,^22^ with a random slope–random intercept to account for clustering by the initial ED (GLMMadaptive: Generalized Linear Mixed Models Using Adaptive Gaussian Quadrature in R version 4.2.2^23^). The unit of analysis was the patient. The model included change categories for ED readiness, patient demographics, comorbidities, initial physiology (Glasgow Coma Scale and age-adjusted hypotension^24^), emergent airway intervention, blood transfusion, mechanism of injury, ISS, transfer status, arrival by ambulance, region, admission year (continuous), and an interaction term (ED readiness change categories by admission year) to allow the slope of pediatric mortality to vary over time for each of the readiness change groups. We estimated the number of additional lives saved vs lost for hospitals in each ED readiness change group using marginal estimated probabilities of mortality from the multivariable model and corresponding SEs from Stata’s MIMRGNS command.^25^ This technique provided estimates for the probability of death under a counterfactual scenario where all EDs had high pediatric readiness, preserving the same distribution of data and controlling for underlying differences.

We calculated 95% CIs using the bootstrap method. We evaluated model fit using the C statistic, tests for multicollinearity, assessment of influential values, and diagnostic plots. Data cleaning and management were performed in SAS statistical software version 9.4 (SAS Institute), and all statistical analyses were performed in R statistical software version 4.2.2 (R Project for Statistical Computing), including a data visualization package,^26^ and Stata statistical software version 18 (StataCorp). All statistical tests were 2-sided with α = .05.

We performed several preplanned sensitivity and subgroup analyses to test the robustness of the findings. For sensitivity analyses, we removed trauma centers with the highest leverage values (most extreme variables), sequentially added hospital-level variables (pediatric ED volume, trauma volume, and trauma level) to the models, and tested a categorical variable for year. We also tested a cutoff point of wPRS greater than or equal to 90 (rather than wPRS ≥93) to define high ED pediatric readiness, representing a midpoint in the wPRS range associated with survival.^5,6,7,8^ The 3 subgroups were based on different definitions of seriously injured children.

Missingness for individual variables ranged from 0% to 9.2% (eTable 2 in Supplement 1). To reduce bias and preserve the study sample, we used multiple imputation to handle missing values,^27,28^ which has been validated for trauma data.^29,30,31,32^ We generated 5 imputed datasets^33^ using multiple imputation via chained equations, as implemented by the MICE package in R^34^ and combined the results with Rubin rules to account for within-dataset and between-dataset variance.^28^

## Results

**Figure 1.**
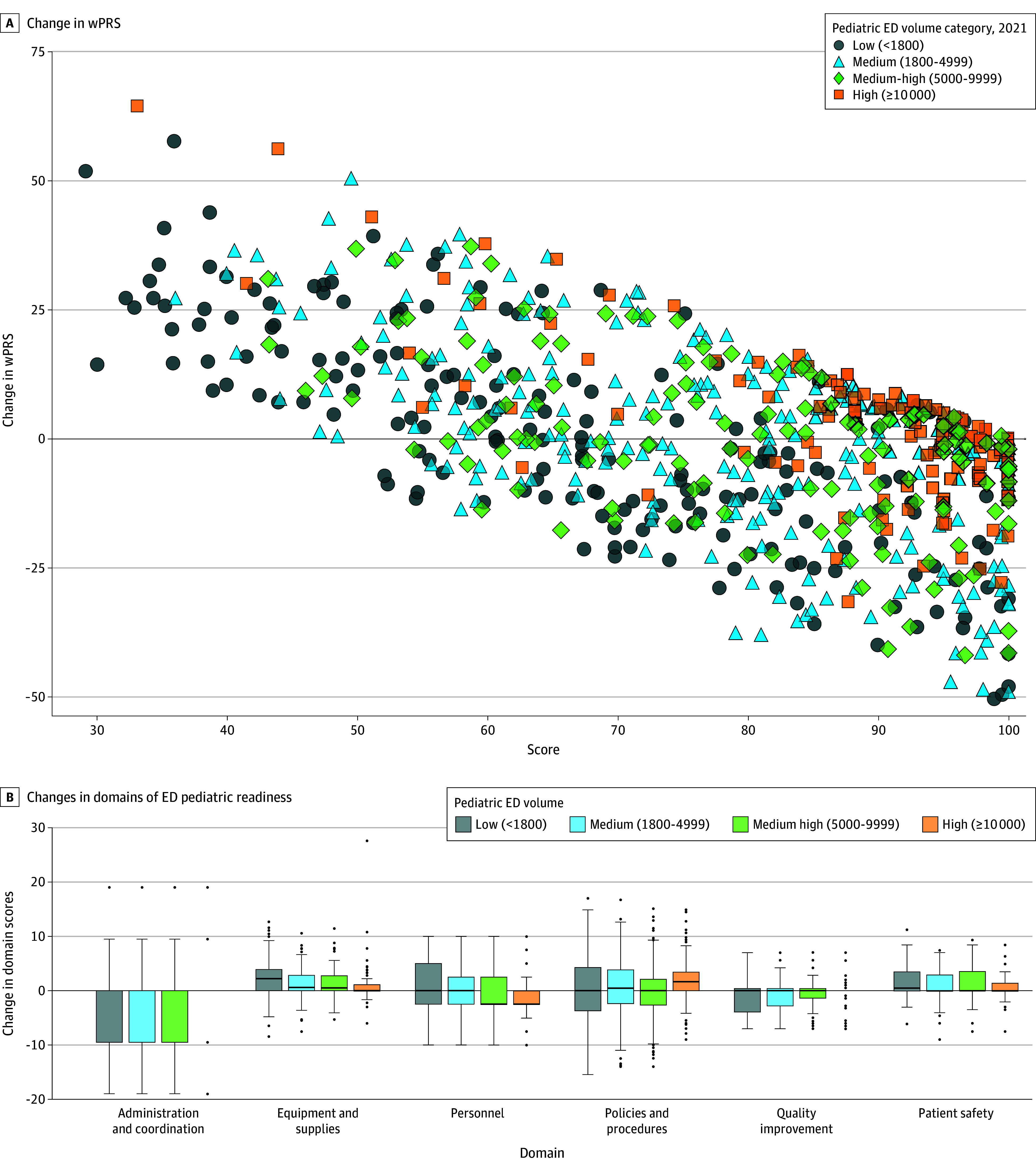
Changes in the Emergency Department (ED) Weighted Pediatric Readiness Score (wPRS) From 2013 to 2021 Among 716 Trauma Centers by Annual ED Pediatric Volume A, Scatterplot shows change in wPRS from 2013 to 2021 by ED pediatric volume. B, Graph shows changes in each of the 6 domains of ED pediatric readiness from 2013 to 2021 by ED pediatric volume. Y-axis represents the absolute change in wPRS from 2013 to 2021. Boxes represent the IQRs, lines in the boxes represent medians, error bars denote 1.5 times the IQR, and dots are outlier values.

There were 467 932 children (300 024 boys [64.1%]; median [IQR] age, 10 [4-15] years; median [IQR] ISS, 4 [4-15]) at 417 trauma centers in the primary analysis, of whom 9544 (2.0%) died (4301 [45.1%] deaths occurred in the ED and 5243 deaths [54.9%] occurred while inpatient). Subgroups included 57 300 children (12.2%) with ISS greater than or equal to 16, 60 927 children (13.0%) with head AIS greater than or equal to 3, and 51 781 children (11.1%) requiring early critical resources. Characteristics of injured children in each of the 4 ED readiness change groups are included in Table 1. Among 716 trauma centers that completed both NPRP assessments, the median (IQR) wPRS decreased from 81 (63-94) in 2013 to 77 (64-93) in 2021, with variability between hospitals (Figure 1A). Readiness scores decreased in the domains of care coordination and quality improvement, while remaining the same or higher in other domains (Figure 1B). The most common category of trauma centers was low-low (440 of 716 centers [61.5%]), which were primarily level III to V trauma centers (246 of 440 centers [55.9%]) that often lacked inpatient pediatric services (eTable 3 in Supplement 1).

**Table 1.** Injured Children Presenting to 417 Trauma Centers, by Quartile of ED Pediatric Readiness Change Group^a^

Characteristic	Children, No. (%) (N = 467 932)			
	Low-low (n = 144 136 [30.8%])	High-low (n = 103 767 [22.2%])	Low-high (n = 64 544 [13.8%])	High-high (n = 155 485 [33.2%])
Age group, y				
0-4	29 123 (20.2)	27 932 (26.9)	16 278 (25.2)	46 115 (29.7)
5-12	42 323 (29.4)	37 889 (36.5)	23 857 (37.0)	59 994 (38.6)
13-15	30 085 (20.9)	19 919 (19.2)	12 433 (19.3)	27 838 (17.9)
16-17	42 605 (29.6)	18 027 (17.4)	11 976 (18.6)	21 538 (13.9)
Sex				
Female	49 339 (34.2)	37 898 (36.5)	23 203 (35.9)	57 468 (37.0)
Male	94 797 (65.8)	65 869 (63.5)	41 341 (64.1)	98 017 (63.0)
Race and ethnicity				
American Indian, Native Hawaiian, or Pacific Islander	15 968 (11.1)	15 958 (15.4)	8811 (13.7)	22 795 (14.7)
Asian	2581 (1.8)	2164 (2.1)	1340 (2.1)	3383 (2.2)
Black	31 105 (21.6)	20 554 (19.8)	13 244 (20.5)	38 245 (24.6)
Hispanic ethnicity	25 084 (17.4)	18 576 (17.9)	13 689 (21.2)	35 903 (23.1)
White	94 482 (65.6)	65 091 (62.7)	41 149 (63.8)	91 062 (58.6)
Mechanism of injury				
Assault or rape	14 119 (9.8)	10 597 (10.0)	6106 (9.5)	14 587 (9.4)
Bicycle	6567 (4.6)	4741 (4.6)	3052 (4.7)	6501 (4.2)
Burn, fire, or flame	3583 (2.5)	2686 (2.6)	1316 (2.0)	5245 (3.4)
Fall	45 568 (31.6)	41 009 (39.5)	24 197 (37.5)	58 180 (37.4)
Gunshot wound	9271 (6.4)	3763 (3.6)	2836 (4.4)	6171 (4.0)
Motor vehicle	31 931 (22.2)	17 134 (16.5)	12 720 (19.7)	28 206 (18.1)
Motorcycle	2061 (1.4)	973 (0.9)	694 (1.1)	1235 (0.8)
Pedestrian	7554 (5.2)	5209 (5.0)	3667 (5.7)	9803 (6.3)
Stabbing, other penetrating injury	5096 (3.5)	3528 (3.4)	1749 (2.7)	4387 (2.8)
Other (eg, blunt object or watercraft)	18 386 (12.8)	14 127 (13.6)	8207 (12.7)	21 170 (13.6)
Transport by ambulance	84 855 (58.9)	58 010 (55.9)	37061 (57.4)	91 551 (58.9)
Initial trauma center level, all types^b^				
Level I	69 331 (48.1)	75 816 (73.1)	40 651 (63.0)	142 285 (91.5)
Level II	53 552 (37.2)	23 454 (22.6)	22 100 (34.2)	13 002 (8.4)
Levels III-V	21 253 (14.8)	4497 (4.3)	1793 (2.8)	198 (0.1)
Initial pediatric trauma center, level I or II^b^	45 780 (31.8)	56 657 (54.6)	48 150 (74.6)	145 848 (93.8)
Rural location of initial trauma center	2260 (2.3)	9902 (9.5)	1141 (1.8)	0
Glasgow Coma Scale score				
3-8 (Worst)	3974 (2.8)	2276 (2.2)	1621 (2.5)	3250 (2.1)
9-12 (Moderate)	9564 (6.6)	6200 (6.0)	3273 (5.1)	9513 (6.1)
13-15 (Best)	130 598 (90.6)	95 291 (91.8)	59 650 (92.4)	142 722 (91.8)
Injury Severity Score				
<9 (Minor)	96 691 (67.1)	70 183 (67.6)	43 115 (66.8)	101 159 (65.1)
9-15 (Moderate)	29 007 (20.1)	21 059 (20.3)	13 428 (20.8)	33 797 (21.7)
16-24 (Serious)	10 358 (7.2)	7697 (7.4)	4843 (7.5)	12 332 (7.9)
≥25 (Severe)	8080 (5.6)	4828 (4.7)	3158 (4.9)	8197 (5.3)
Serious head injury (Abbreviated Injury Scale head and neck ≥3)	17 692 (12.3)	14 436 (13.9)	8939 (13.8)	22 545(14.5)
Airway or ventilation support	11 878 (8.2)	7031 (6.8)	4870 (7.5)	11 895 (7.7)
Blood transfusion	6302 (4.4)	3648 (3.5)	2242 (3.5)	5333 (3.4)
Nonorthopedic surgery	13 361 (9.3)	8470 (8.2)	5595 (8.7)	14 136 (9.1)
Orthopedic surgery	45 208 (31.4)	37 744 (36.4)	24 187 (37.5)	58 018 (37.3)
Transfer to another trauma center	9113 (6.3)	1642 (1.6)	1058 (1.6)	659 (0.4)
Length of stay, mean (SD), d	2.4 (6.0)	2.5 (13.2)	2.4 (5.9)	2.6 (6.2)
ED and in-hospital mortality	3838 (2.7)	1804 (1.7)	1288 (2.0)	2614 (1.7)

Abbreviation: ED, emergency department.

^a^ The ED pediatric readiness change groups are based on assessments in 2013 and 2021, defining high readiness as a weighted pediatric readiness score (wPRS; range, 0-100) 93 or higher and low readiness as wPRS less than 93.

^b^ Trauma center level and type were based on 2021 information from the National Trauma Data Bank and the national ED pediatric readiness assessments. Because we combined trauma center type (adult, pediatric, mixed) to generate trauma level, we used the highest-ranking level (eg, level I) for trauma centers that had differing designations for adult vs pediatric trauma center status.

Among the 417 trauma centers with patient-level data, observed (unadjusted) mortality was lower in the high-high (2614 deaths among 155 485 children [1.7%]), low-high (1288 deaths among 64 544 children [2.0%]), and high-low (1804 deaths among 103 767 children [1.7%]) change groups compared with the low-low group (3838 deaths among 144 136 children [2.7%]), but other comparisons were inconsistent (Table 1). After risk adjustment, there was an independent association between high ED pediatric readiness and low mortality in 2012 to 2014 (adjusted odds ratio [aOR] highest vs lowest quartile of ED readiness, 0.50; 95% CI, 0.34-0.74; 138 333 children) and 2019 to 2021 (aOR, 0.64; 95% CI, 0.47-0.87; 142 199 children). Results from the multivariable model using data across the 10-year period (2012-2021) are presented in Table 2. On the basis of the interaction term, trauma centers in the high-low group had an increased odds of death each year (aOR high-low vs high-high, 1.08; 95% CI, 1.01-1.15). A similar association among trauma centers in the low-low group did not reach statistical significance in the primary model (aOR low-low vs high-high, 1.05; 95% CI, 0.99-1.11), but was evident after adjusting for trauma level (aOR, 1.06; 95% CI, 1.00-1.12) (eTable 4 in Supplement 1). Results were similar after accounting for pediatric volume (eTable 4 in Supplement 1) and after removing outlier trauma centers (eTable 5 in Supplement 1).

**Table 2.** Multivariable Model of Changes to ED Pediatric Readiness Over Time (2012-2021) and Mortality for 467 932 Injured Children in 417 Trauma Centers

Variable	ED and in-hospital mortality, aOR (95% CI)^a^
ED readiness change group, main effect size^b^	
Low-low	1.38 (0.99-1.94)
High-low	0.93 (0.61-1.40)
Low-high	1.25 (0.80-1.95)
High-high	1 [Reference]
Year of visit, main effect size^c^	0.95 (0.91-1.00)
Interaction of ED readiness change group with year^d^	
Low-low by year	1.05 (0.99-1.11)
High-low by year	1.08 (1.01-1.15)
Low-high by year	1.02 (0.95-1.10)
High-high by year	1 [Reference]
Female sex	0.99 (0.92-1.07)
Age group, y	
0-4	1.66 (1.48-1.85)
5-12	1.10 (0.99-1.23)
13-15	1.00 (0.90-1.11)
16-17	1 [Reference]
Race	
Asian	1.06 (0.81-1.38)
Black	0.93 (0.85-1.02)
Other^e^	1.04 (0.93-1.17)
White	1 [Reference]
Comorbid conditions	
0	1 [Reference]
1	0.78 (0.70-0.88)
≥2	0.23 (0.21-0.25)
Injury mechanism	
Fall	1 [Reference]
Gunshot wound	8.04 (6.78-9.53)
Stabbing or other penetrating injury	2.09 (1.52-2.87)
Burn, fire, or flame	4.65 (3.43-6.30)
Assault	1.71 (1.36-2.14)
Motor vehicle	1.42 (1.22-1.65)
Bicycle	1.43 (1.09-1.86)
Pedestrian vs automobile	1.86 (1.58-2.20)
Motorcycle	1.47 (1.09-1.98)
Other	2.76 (2.35-3.23)
Arrival by ambulance	1.78 (1.50-2.10)
Transfer to another hospital	0.35 (0.28-0.44)
Age-adjusted hypotension	14.09 (12.96-15.32)
Glasgow Coma Scale score	
≤8 (Worst)	28.84 (25.36-32.81)
9-12 (Moderate)	12.53 (11.04-14.23)
≥13 (Best)	1 [Reference]
Injury Severity Score	
<9 (Minor)	1 [Reference]
9-15 (Moderate)	6.33 (4.97-8.06)
16-24 (Serious)	6.92 (5.44-8.79)
≥25 (Severe)	28.80 (22.79-36.39)
Airway or ventilation support	21.66 (18.28-25.65)
Blood transfusion (any)	1.66 (1.53-1.80)

Abbreviations: aOR, adjusted odds ratio; ED, emergency department.

^a^ The model also included Northeast, South, West, and Midwest regions (data not shown).

^b^ The ED readiness change groups were defined by a high weighted pediatric readiness score (wPRS; ≥93) vs a low wPRS (<93) in 2013 vs 2021, creating 4 groups: high-high (wPRS ≥93 during both assessments), low-high (wPRS <93 in 2013 and wPRS ≥93 in 2021), high-low (wPRS ≥93 in 2013 and <93 in 2021), and low-low (wPRS <93 during 2013 and 2021). Because the ED readiness change group variable was included in an interaction with year, aORs for the main effect size of the ED readiness change group variable represent the odds of mortality during the first year of the study (2012). aORs greater than 1 for the ED readiness change group indicate that the readiness group had higher adjusted odds of mortality during the first year of the study compared with the referent group (high-high).

^c^ Year was modeled as a continuous variable. Because year was included in the interaction term with ED readiness change group, the aOR for year (main effect size) indicates the yearly odds of mortality for the referent group of ED readiness (high-high). An aOR less than 1 for year indicates that the high-high ED readiness change group had a yearly decrease in odds of mortality.

^d^ For the interaction term (ED readiness change group by year of visit), the aORs indicate the yearly change in odds of mortality for each readiness change group compared with the referent readiness group (high-high). For example, compared with high-high EDs, high-low EDs had a yearly aOR of 1.08 (95% CI, 1.01-1.15). In other words, high-low EDs had 8% increased odds of mortality each year compared with high-high EDs.

^e^ The other category for race included American Indian, Native Hawaiian, and Pacific Islander.

**Figure 2.**
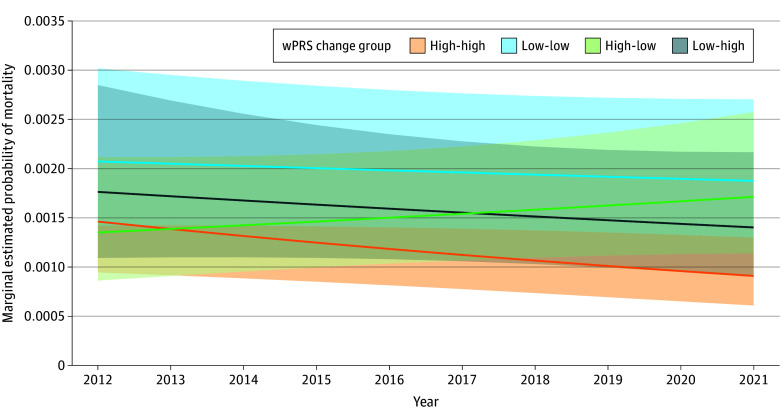
Adjusted In-Hospital Mortality Over Time Among Trauma Centers With 4 Patterns of Emergency Department (ED) Pediatric Readiness Over Time (N = 467 932) The 4 ED readiness change groups included high-high (weighted pediatric readiness score [wPRS], ≥93 during both assessments), low-high (wPRS <93 in 2013 and ≥93 in 2021), high-low (wPRS ≥93 in 2013 and <93 in 2021), and low-low (wPRS <93 during 2013 and 2021). The shaded areas denote 95% CIs.

The marginal estimated probability of death over the study period differed for each of the ED readiness change groups, with overlap in 95% CIs (Figure 2). Adjusted mortality was lowest among trauma centers with persistently high ED pediatric readiness, whereas it was highest for hospitals with persistently low ED readiness. Mortality increased over time among trauma centers that changed from high to low ED readiness, whereas trauma centers that changed from low to high ED readiness had the opposite trend. Findings were similar in subgroup analyses (eTable 6 and eFigure 2 in Supplement 1) and when using a categorical variable for year (eFigure 3 in Supplement 1).

Over the 10-year period and using wPRS greater than or equal to 93 to define high ED pediatric readiness, trauma centers that had high readiness throughout the study period or that moved from low to high readiness had 643 additional lives saved (95% CI, −328 to 1599 lives saved) (Table 3). Conversely, trauma centers with persistently low ED readiness or that changed from high to low readiness had 729 additional lives lost (95% CI, −373 to 1831 lives lost) (Table 3). When using a threshold of wPRS 90 or higher, hospitals with high readiness or moving from low to high readiness showed 973 lives saved (95% CI, −499 to 2445 lives saved), whereas hospitals with low readiness or moving from high to low readiness showed 618 lives lost (95% CI, 1 to 1235 lives lost) (Table 3).

**Table 3.** Numbers of Lives Saved vs Lost Due to Changes to ED Pediatric Readiness Between 2013 and 2021 Using Different Thresholds to Define High ED Readiness in 417 Trauma Centers

Variable	Lives, No. (95% CI) (N = 467 932)^a^
Using wPRS ≥93 to define high ED pediatric readiness	
Lives saved due to high readiness	
High-high EDs (64 EDs; 156 523 patients); persistently high level of ED pediatric readiness (vs low-low)	543 (−350 to 1438)
Low-high EDs (54 EDs; 68 422 patients); increased level of ED pediatric readiness over time (vs low-low)	100 (−189 to 386)
Total lives saved due to high readiness	643 (−328 to 1599)
Lives lost due to low readiness	
High-low EDs (66 EDs; 105 911 patients); decreased level of ED pediatric readiness over time (vs high-high)	160 (−353 to 662)
Low-low EDs (233 EDs; 151 382 patients); persistently low level of ED pediatric readiness (vs high-high)	569 (−287 to 1435)
Total lives lost due to low readiness	729 (−373 to 1831)
Net change in pediatric lives related to changes in ED pediatric readiness, lives lost	86 (−1377 to 1547)
Using wPRS ≥90 to define high ED pediatric readiness	
Lives saved due to high readiness	
High-high EDs (95 EDs; 222 966 patients); persistently high level of ED pediatric readiness (vs low-low)	768 (−651 to 2184)
Low-high EDs (61 EDs; 63 040 patients); increased level of ED pediatric readiness over time (vs low-low)	205 (−28 to 442)
Total lives saved due to high readiness	973 (−499 to 2445)
Lives lost due to low readiness	
High-low EDs (71 EDs; 91 734 patients); decreased level of ED pediatric readiness over time (vs high-high)	220 (−181 to 614)
Low-low EDs (190 EDs; 104 499 patients); persistently low level of ED pediatric readiness (vs high-high)	398 (−68 to 866)
Total lives lost due to low readiness	618 (1 to 1235)
Net change in pediatric lives related to changes in ED pediatric readiness, lives saved	355 (−1213 to 1923)

Abbreviations: ED, emergency department; wPRS, weighted pediatric readiness score.

^a^ Sample sizes for each category were calculated as if each of the 417 trauma centers provided all 10 years (2012-2021) of data.

Model diagnostics indicated that random intercept–random slope models were superior to models using fixed effects and random intercept alone. The final model did not have evidence of multicollinearity and had a C statistic of 0.994. Calibration plots did not show deviations between estimated and observed values.

## Discussion

This cohort study found that ED pediatric readiness decreased in US trauma centers between 2013 and 2021, particularly owing to the loss of PECCs and reduction in quality improvement. Observed mortality was highest in EDs with persistently low pediatric readiness, but fluctuated among other readiness change groups. Our risk-adjusted findings suggested that high ED pediatric readiness at trauma centers (persistent or change to) was associated with pediatric lives saved after injury. Conversely, low ED readiness at trauma centers was associated with lives lost. Although the findings were not statistically significant, the results suggest that changes to ED pediatric readiness over time are associated with pediatric lives saved and lives lost, depending on the direction of change. To our knowledge, this study is the first to evaluate the mortality associated with changes in ED pediatric readiness over the past decade among US trauma centers.

Our findings have implications for national trauma policy. First, there was a robust association between high ED pediatric readiness and low mortality across both time periods, showing the persistent benefit of ED readiness at US trauma centers. Next, the trends in adjusted mortality over time among ED readiness change groups followed an expected pattern, with improved ED readiness associated with reduced mortality and decreased ED readiness associated with increased mortality. The preventable mortality associated with these shifts was measurable, highlighting the importance of ED pediatric readiness in trauma center verification.^9^ Finally, although previous research^5,6,7,8^ has shown that different wPRS thresholds are associated with survival, our results suggest that wPRS 90 or higher may be an appropriate target to optimize pediatric survival in trauma centers. Currently, there is no minimum threshold of wPRS expected for trauma centers.^9^ Although the association between individual components of readiness and survival has differed across studies,^35,36^ the first step to improving ED pediatric readiness is adding PECCs, who are integral to all aspects of ED readiness^37^ and are typically existing ED nurses and physicians.^38,39^ There are publicly available checklists and toolkits designed to assist EDs in increasing their level of pediatric readiness.^40^

ED pediatric readiness can play a role in reversing the current trend of increasing mortality among children in the US (especially from injury).^10^ Although injury prevention efforts are an integral aspect of a multifactorial solution, increasing ED pediatric readiness is an actionable goal that can be implemented immediately. Our results complement research showing that increasing the level of ED readiness among all trauma centers would more than triple the number of pediatric lives saved compared with selective transport to high-readiness trauma centers.^41^ Because only one-half of children in the US reside within 1 hour of a pediatric trauma center (most of which have high ED readiness) by ground transport,^42^ there is an urgent need for all trauma centers to boost efforts to serve injured children.

### Limitations

There are limitations to our study. The sample included trauma centers with NTDB participation over a 10-year period that completed both ED readiness assessments, representing hospitals with greater resources, trauma infrastructure, and a commitment to quality improvement. Despite efforts to be inclusive with trauma centers for the primary analysis, the sample was likely biased toward higher-performing hospitals, which limited the statistical power to show differences. Therefore, our results provide conservative estimates of the preventable mortality associated with changes to ED pediatric readiness and are not statistically conclusive. In addition, we created a dichotomous definition for high vs low ED readiness to facilitate comparisons between the readiness change groups. Although this definition is supported by previous research,^5,6,7,8^ it grouped a large number of hospitals with varying levels of ED readiness into the low group, which may have further reduced statistical power.

Because there were only 2 assessments of ED pediatric readiness, it was not possible to know exactly when changes occurred in trauma centers. We suspect that many of the reductions in ED readiness occurred from 2020 to 2021 in the context of other shifts in staffing and resources related to the COVID-19 pandemic. However, shifts in pediatric care among US hospitals began well before the pandemic, including a large reduction in pediatric inpatient services, especially in rural hospitals.^43,44^ If changes in ED readiness occurred late in the study period, with less exposure of pediatric patients to the changes, the association with mortality may have been diminished. Furthermore, the statistical models assumed linear changes to mortality over time in the ED readiness change groups, but the association may be nonlinear. However, results using a categorical variable that allowed year-to-year variation in mortality were similar.

## Conclusions

ED pediatric readiness declined among US trauma centers over the past decade, with variability between hospitals. These changes were associated with mortality, lives saved, and lives lost, depending on the direction of change. Many US trauma centers are not yet optimized for pediatric survival after injury. With ED pediatric readiness integrated to the updated trauma center verification criteria, our findings provide further impetus for trauma centers to increase their level of pediatric readiness.

## Notes

Supplement 1. **eFigure 1.** Schematic of cohort creation **eTable 1.** Measures of early resource need **eTable 2.** Missingness of variables **eTable 3.** Hospital characteristics by wPRS change group of emergency department pediatric readiness (n = 716 trauma centers) **eTable 4.** Multivariable model results based on the sequential addition of hospital-level variables (n = 467,932) **eTable 5.** Multivariable model after dropping outlying trauma centers (n = 465,068) **eTable 6.** Multivariable model results from subgroup analyses **eFigure 2.** Subgroup results for the adjusted predicted probability of death for the ED pediatric readiness change groups **eFigure 3.** Adjusted in-hospital mortality over time among trauma centers with four patterns of emergency department pediatric readiness and using a categorical variable for year (n = 467,932)

Supplement 2.
Data Sharing Statement
