# Supplementary material for: Changes in Emergency Department Pediatric Readiness and Mortality
Source: JAMA Netw Open. 2024 Jul 22;7(7):e2422107. doi: 10.1001/jamanetworkopen.2024.22107 (PMC11265139; doi:10.1001/jamanetworkopen.2024.22107)
Supplement: Supplement 1. — eFigure 1. Schematic of cohort creation eTable 1. Measures of early resource need eTable 2. Missingness of variables eTable 3. Hospital characteristics by wPRS change group of emergency department pediatric readiness (n = 716 trauma centers) eTable 4. Multivariable model results based on the sequential addition of hospital-level variables (n = 467,932) eTable 5. Multivariable model after dropping outlying trauma centers (n = 465,068) eTable 6. Multivariable model results from subgroup analyses eFigure 2. Subgroup results for the adjusted predicted probability of death for the ED pediatric readiness change groups eFigure 3. Adjusted in-hospital mortality over time among trauma centers with four patterns of emergency department pediatric readiness and using a categorical variable for year (n = 467,932) [file jamanetwopen-e2422107-s001.pdf]

Supplemental Online Content

Newgard CD, Rakshe S, Salvi A, et al. Changes in emergency department pediatric readiness and mortality. *JAMA Netw Open*. 2024;7(7):e2422107.
doi:10.1001/jamanetworkopen.2024.22107

eFigure 1. Schematic of cohort creation

eTable 1. Measures of early resource need

eTable 2. Missingness of variables

eTable 3. Hospital characteristics by wPRS change group of emergency department pediatric readiness (n = 716 trauma centers)

eTable 4. Multivariable model results based on the sequential addition of hospital-level variables (n = 467,932)

eTable 5. Multivariable model after dropping outlying trauma centers (n = 465,068)

eTable 6. Multivariable model results from subgroup analyses

eFigure 2. Subgroup results for the adjusted predicted probability of death for the ED pediatric readiness change groups

eFigure 3. Adjusted in-hospital mortality over time among trauma centers with four patterns of emergency department pediatric readiness and using a categorical variable for year (n = 467,932)

This supplemental material has been provided by the authors to give readers additional information about their work.

© 2024 Newgard CD et al. *JAMA Network Open.*

eFigure 1. Schematic of cohort creation.

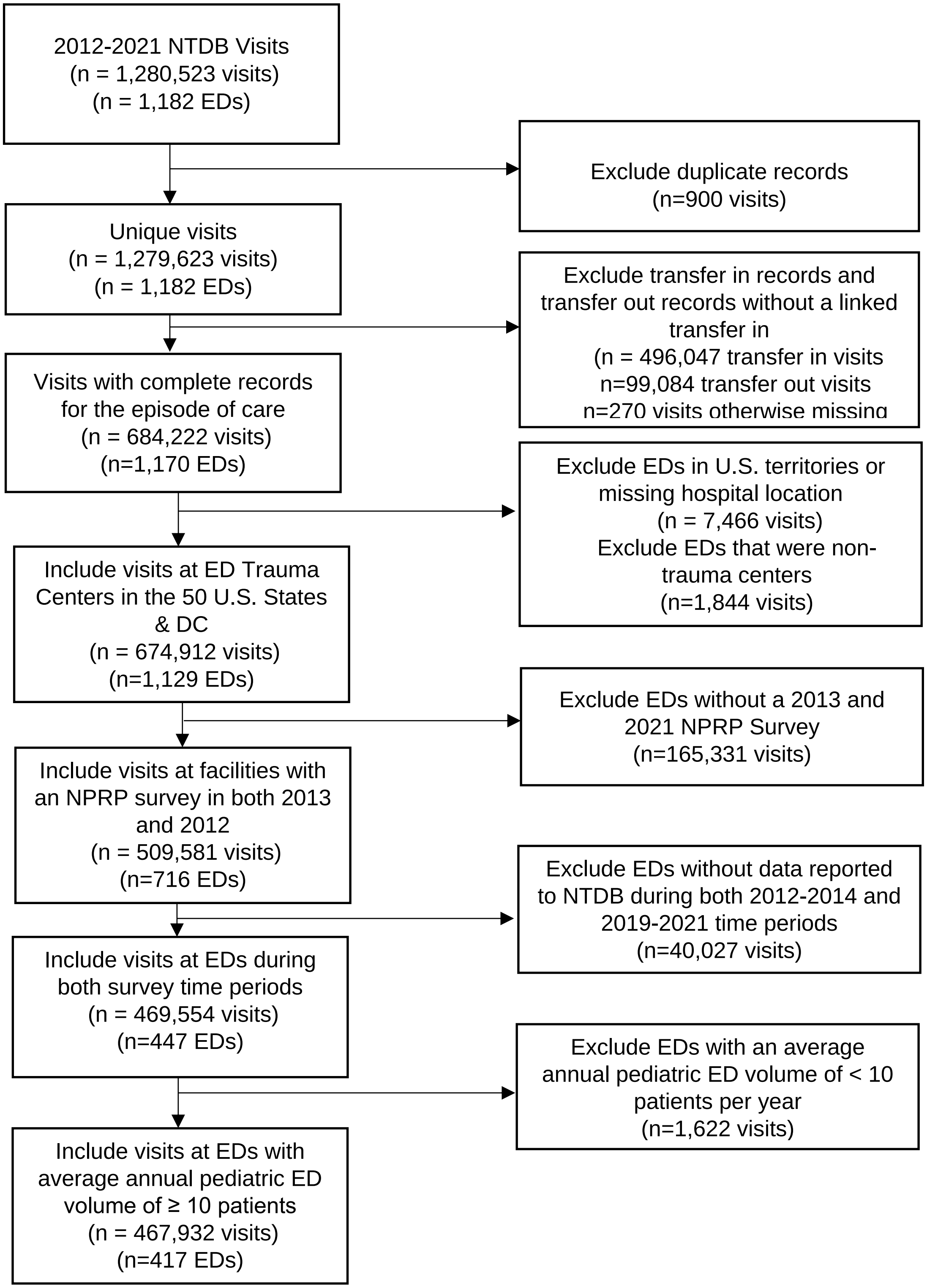

© 2024 Newgard CD et al. *JAMA Network Open.*

eTable 1. Measures of early resource need

Resource	n	(% of sample)
Any early resource need	**51,781**	**(11.1%)**
Advanced airway management	34,664	(7.4%)
Blood transfusion	17,525	(3.8%)
Brain procedure	11,403	(2.4%)
Cardiac procedure	4023	(0.9%)
Neck or spine procedures	1737	(0.4%)
Thoracic procedure	12,617	(2.7%)
Abdominal procedure	19,823	(4.2%)
Vascular procedure	5529	(1.2%)
Vasopressor support	510	(0.1%)
Extra corporeal membrane oxygenation	142	(<0.1%)
Presence of spinal cord injury	8357	(1.8%)

© 2024 Newgard CD et al. *JAMA Network Open.*

eTable 2. Missingness of variables

Variable	N=467,932 (100%)
Systolic Blood Pressure	43172 (9.2)
Ethnicity	40264 (8.6)
Respiratory Rate	29201 (6.2)
Glasgow Coma Scale	27035 (5.8)
Race	16366 (3.5)
Mechanism of injury	15735 (3.4)
Upper lower extremity AIS	11035 (2.4)
Abdomen AIS	10689 (2.3)
Thorax AIS	8910 (1.9)
Head and neck AIS	7851 (1.7)
Transport by ambulance	7371 (1.6)
Injury Severity Score	5949 (1.3)
Length of stay	4616 (0.9)
Emergency airway intervention	1089 (0.2)
Gender	1080 (0.2)

Abbreviations: AIS, Abbreviated Injury Scale

© 2024 Newgard CD et al. *JAMA Network Open.*

eTable 3. Hospital characteristics by wPRS change group of emergency department pediatric readiness (n = 716 trauma centers)

	WPRS Change Group			
**Measure**	**Low-Low n = 440** (61%)	**High-Low n = 93** (13%)	**Low-High n = 89** (12%)	**High-High n = 94** (13%)
**Pediatric Ward**				
Yes	220 (50%)	65 (70%)	70 (79%)	82 (87%)
**Pediatric ICU unit**				
Yes	60 (14%)	45 (48%)	42 (47%)	72 (77%)
**Does the hospital transfer most severely injured children to other specialty centers?**				
Yes	404 (92%)	56 (60%)	62 (70%)	35 (37%)
**Geographic region**				
Northeast	36 (8%)	10 (11%)	14 (16%)	8 (8.5%)
Midwest	130 (30%)	26 (28%)	33 (37%)	29 (31%)
South	158 (36%)	34 (37%)	21 (24%)	35 (37%)
West	116 (26%)	23 (25%)	21 (24%)	22 (23%)
**Hospital geographic location**				
Rural/remote	96 (21.8%)	8 (8.6%)	5 (5.6%)	0 (0%)
Suburban	27 (6.1%)	2 (2.2%)	0 (0%)	1 (1.1%)
Urban	317 (72.1%)	83 (89.3%)	84 (94.4%)	93 (98.9%)
**Hospital teaching status**				
Non-teaching	185 (42%)	25 (27%)	19 (21%)	14 (15%)
Community	192 (44%)	43 (46%)	45 (51%)	31 (33%)
University	63 (14%)	25 (27%)	25 (28%)	49 (52%)
**Initial trauma center level, all types**				
Level 1	66(15%)	33(35%)	33(37%)	62(66%)
Level 2	128(29%)	38(41%)	37(42%)	24(26%)
Level 3/4/5	246(56%)	22(24%)	19(21%)	8(9%)
**Initial pediatric trauma center**				
Level 1 or 2	38(9%)	29(31%)	37(42%)	60(64%)
**Annual ED pediatric patient volume**				
Low (<1,800)	162(37%)	17(18%)	6(7%)	3(3%)
Medium (1,800-4,999)	157(36%)	30(32%)	34(38%)	16(17%)
Medium High (5,000-9,999)	87(20%)	18(19%)	19(21%)	15(16%)
High (≥10,000)	34(8%)	28(30%)	30(34%)	60(64%)

© 2024 Newgard CD et al. *JAMA Network Open.*

eTable 4. Multivariable model results based on the sequential addition of hospital-level variables (n = 467,932)

**Primary model + annual ED pediatric volume**	**OR (95%CI)**
ED Readiness Change Group, Main effects[1]	
Low-Low	1.32(0.93-1.87)
High-Low	0.89(0.59-1.36)
Low-High	1.19(0.76-1.87)
High-High	referent
Year of visit	0.95(0.91-1.00)
ED Readiness Change Group by Year, Interaction effects[2]	
Low-Low by Year	1.05(0.99-1.11)
High-Low by Year	1.08(1.01-1.16)
Low-High by Year	1.03(0.96-1.10)
High-High by Year	Referent
**Primary model + annual pediatric trauma admission volume**	**OR (95%CI)**
ED Readiness Change Group, Main effects[1]	
Low-Low	1.08(0.77-1.51)
High-Low	0.84(0.56-1.27)
Low-High	1.10(0.71-1.72)
High-High	referent
Year of visit	0.96(0.92-1.00)
ED Readiness Change Group by Year, Interaction effects[2]	
Low-Low by Year	1.04(0.99-1.10)
High-Low by Year	1.08(1.01-1.15)
Low-High by Year	1.03(0.96-1.10)
High-High by Year	referent
**Primary model + trauma center level** (Level I, II, or III/IV/V)	**OR (95%CI)**
ED Readiness Change Group, Main effects[1]	
Low-Low	1.07(0.76-1.49)
High-Low	0.78(0.52-1.17)
Low-High	1.06(0.68-1.64)
High-High	referent
Year of visit	0.95(0.91-1.00)
ED Readiness Change Group by Year, Interaction effects[2]	
Low-Low by Year	1.06(1.00-1.12)
High-Low by Year	1.08(1.01-1.16)
Low-High by Year	1.03(0.96-1.11)
High-High by Year	referent

© 2024 Newgard CD et al. *JAMA Network Open.*

eTable 5. Multivariable model after dropping outlying trauma centers (n = 465,068)

**Primary model, removing top 10 outlying EDs n = 465,068**	**OR (95%CI)**
ED Readiness Change Group, Main effects[1]	
Low-Low	1.43(1.02-2.01)
High-Low	0.94(0.62-1.42)
Low-High	1.25(0.80-1.96)
High-High	referent
Year of visit[2]	0.95(0.91-0.99)
ED Readiness Change Group by Year, Interaction effects[3]	
Low-Low by Year	1.05(0.99-1.10)
High-Low by Year	1.08(1.01-1.15)
Low-High by Year	1.02(0.95-1.10)
High-High by Year	referent

© 2024 Newgard CD et al. *JAMA Network Open.*

eTable 6. Multivariable model results from subgroup analyses

**Injury Severity Score ≥ 16, n =57,300**	**OR (95%CI)**
ED Readiness Change Group, Main effects[1]	
Low-Low	1.50 (1.19-1.89)
High-Low	1.05 (0.79-1.38)
Low-High	1.25 (0.92-1.69)
High-High	referent
Year of visit	0.97 (0.94-1.00)
ED Readiness Change Group by Year, Interaction effects[2]	
Low-Low by Year	1.02 (0.98-1.06)
High-Low by Year	1.05 (1.00-1.10)
Low-High by Year	1.02 (0.96-1.07)
High-High by Year	referent
**Serious Head Injury (head AIS ≥ 3), n =60,927**	
ED Readiness Change Group, Main effects[1]	
Low-Low	1.49 (1.16-1.90)
High-Low	1.06 (0.79-1.42)
Low-High	1.14 (0.83-1.57)
High-High	referent
Year of visit	0.95 (0.92-0.98)
ED Readiness Change Group by Year, Interaction effects[2]	
Low-Low by Year	1.03 (0.99-1.08)
High-Low by Year	1.05 (1.00-1.10)
Low-High by Year	1.04 (0.98-1.10)
High-High by Year	referent
**Need For Early Critical Resources, n =51,781**	
ED Readiness Change Group, Main effects[1]	
Low-Low	1.51 (1.18-1.92)
High-Low	1.05 (0.79-1.40)
Low-High	1.26 (0.92-1.73)
High-High	referent
Year of visit	0.98 (0.94-1.01)
ED Readiness Change Group by Year, Interaction effects[2]	
Low-Low by Year	1.02 (0.98-1.06)
High-Low by Year	1.04 (0.99-1.10)
Low-High by Year	1.01 (0.96-1.07)
High-High by Year	referent

© 2024 Newgard CD et al. *JAMA Network Open.*

eFigure 2. Subgroup results for the adjusted predicted probability of death for the ED pediatric readiness change groups

A. Injury Severity Score ≥ 16 (n = 57,300).

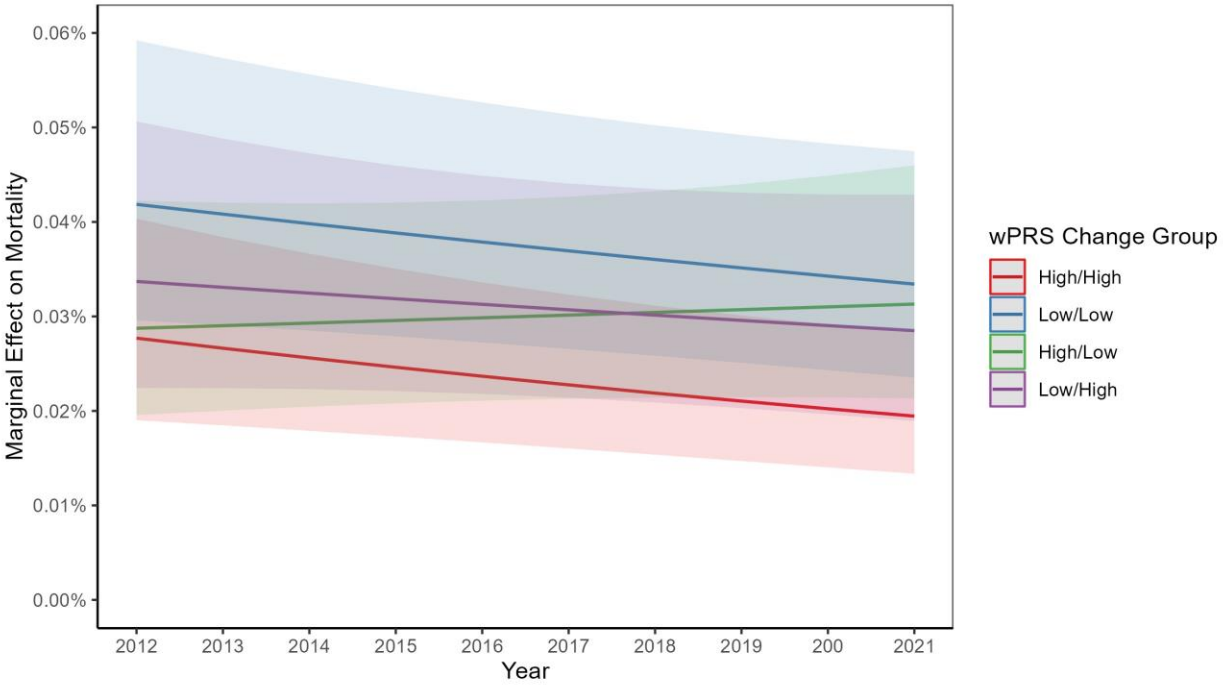

B. Serious head injury, Abbreviated Injury Scale ≥ 3 (n = 60,927).

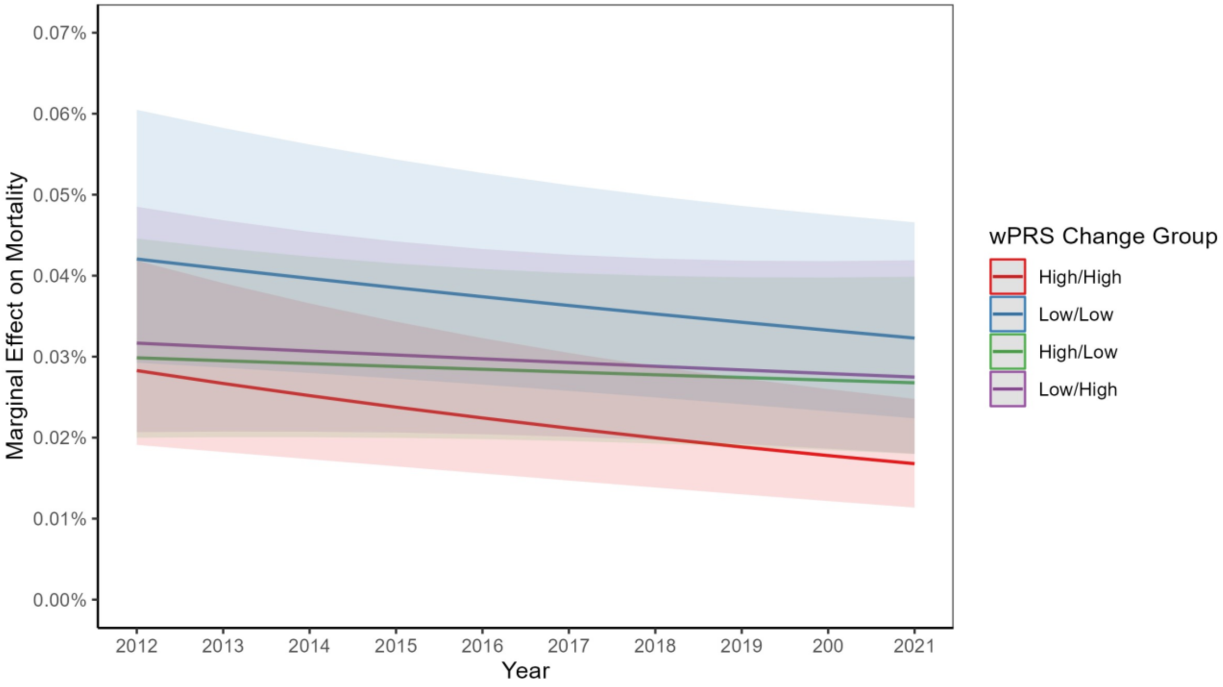

© 2024 Newgard CD et al. *JAMA Network Open.*

C. Need for early critical resources (n = 51,781).

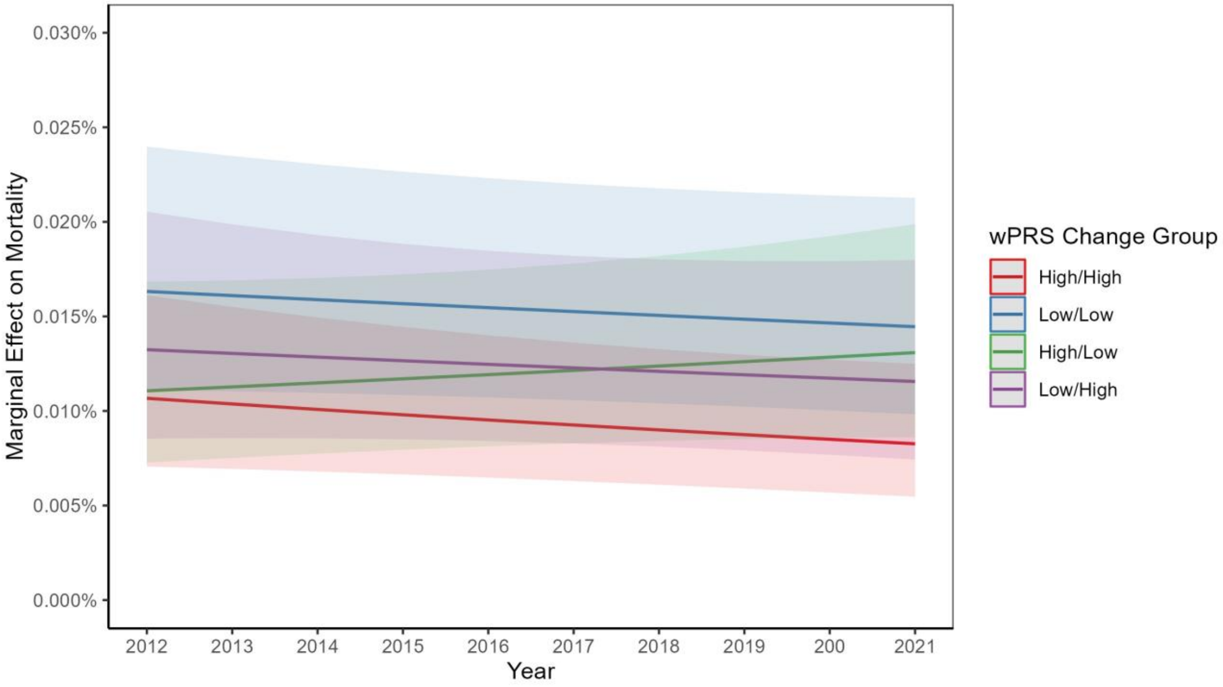

© 2024 Newgard CD et al. *JAMA Network Open.*

eFigure 3. Adjusted in-hospital mortality over time among trauma centers with four patterns of emergency department pediatric readiness and using a categorical variable for year (n = 467,932)

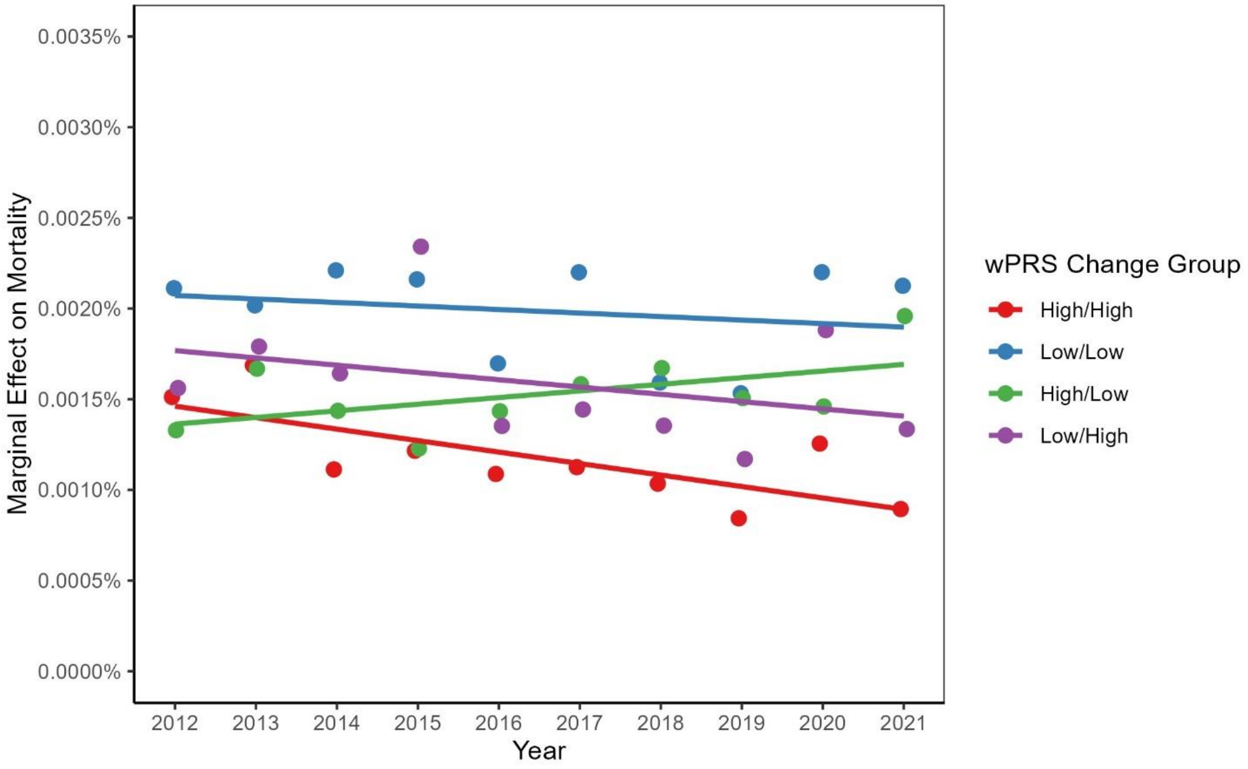

*The risk-adjustment model used to generate the figure is identical to that used for Figure 2, including the interaction of ED readiness change group × year, except that year was modeled as a categorical variable and allowed to vary over each year. The lines represent the best-fit linear slope for each of the four ED readiness change groups.

© 2024 Newgard CD et al. *JAMA Network Open.*
