# Supplementary material for: Changes in Emergency Department Pediatric Readiness and Mortality
Source: JAMA Netw Open. 2024 Jul 22;7(7):e2422107. doi: 10.1001/jamanetworkopen.2024.22107 (PMC11265139; doi:10.1001/jamanetworkopen.2024.22107)
Supplement: Supplement 2. — Data Sharing Statement [file jamanetwopen-e2422107-s002.pdf]

Data Sharing Statement

Newgard. Changes in Emergency Department Pediatric Readiness and Mortality. *JAMA Netw Open*. Published July 15, 2024. doi:10.1001/jamanetworkopen.2024.22107

Data
Data available: No

Additional Information
Explanation for why data not available: Due to existing DUAs, we are not able to share these data. However, they are available from the American College of Surgeons and the EMS for Children Data Center (University of Utah).
